# Virtual Reality Assessment of Classroom – Related Attention: An Ecologically Relevant Approach to Evaluating the Effectiveness of Working Memory Training

**DOI:** 10.3389/fpsyg.2019.01851

**Published:** 2019-08-20

**Authors:** Benjamin Coleman, Sarah Marion, Albert Rizzo, Janiece Turnbull, Anne Nolty

**Affiliations:** ^1^College of Extended Learning, Point Loma Nazarene University, San Diego, CA, United States; ^2^Northwest Nazarene University, Nampa, ID, United States; ^3^Institute of Creative Technologies, University of Southern California, Los Angeles, CA, United States; ^4^Fuller Graduate School of Psychology, Pasadena, CA, United States

**Keywords:** virtual reality, ADHD, cognitive training and brain training, ecological validity, working memory

## Abstract

Computerized cognitive interventions to improve working memory also purport to improve ADHD-related inattention and off task behavior. Such interventions have been shown to improve working memory, executive functioning, and fluid reasoning on standardized neuropsychological measures. However, debate continues as to whether such programs lead to improvement on ecologically relevant outcomes, such as classroom behavior. This study sought to propose a novel, ecologically relevant approach to evaluate the effectiveness of working memory training on real-world attention performance. Participants included 15 children, aged 6–15, identified as having attention problems were assessed via the virtual classroom continuous performance task (VCCPT) before and after completing 5 weeks of Cogmed working memory training. The VCCPT is a validated measure of sustained and selective attention set within a virtual reality (VR) environment. Several key areas of attention performance were observed to improve, including omission errors, reaction time, reaction time variability, and hit variability. Results suggest that working memory training led to substantial improvements in sustained attention in a real-life scenario of classroom learning. Moreover, the use of psychometrically validated VR measurement provides incremental validity beyond that of teacher or parent report of behavior. Observing such improvements on ecologically relevant measures of attention adds to the discussion around how to evaluate the effectiveness of working memory training as it pertains to real-life improvements and serves to inform consumer awareness of such products and their claims.

## Introduction

Virtual reality (VR) provides an exciting medium in which to gather evidence for ecological relevance that goes beyond traditional cognitive assessment (e.g., Schultheis and Rizzo, 2001; Rizzo and Koenig, 2017), enabling researchers to observe behaviors in simulated real life settings with participants less aware of the examiner while immersed in the simulated environment (Draeger et al., 1986; Baumgartner et al., 2008). VR assessment also provides increased levels of experimental control compared to traditional pencil and paper methods (Rizzo et al., 2004). The VR head-mounted display tracks and records the participants’ head movements, allowing for more objective behavioral assessment of off-task behavior and hyperactivity (Rizzo et al., 2006).

### The Virtual Classroom

Continuous performance tests (CPTs) typically show group differences between individuals with ADHD and control participants (Denney et al., 2005). However, the sensitivity of traditional CPTs to identify distractibility has been criticized (Adams et al., 2009). In response, the virtual classroom (Rizzo et al., 2000), a CPT embedded within a virtual environment, depicts a real-life setting more accurately than traditional designs. In this virtual scenario, the user must respond to stimuli as viewed on the chalkboard from his or her desk chair while resisting distractions typical of an academic setting (e.g., peers throwing paper airplanes, whispering, and windows through which traffic can be seen and heard). Further, the virtual classroom apparatus allows for the measurement of head movements during the task, a variable shown to be associated with hyperactivity (Teicher, 1996). Research using the virtual classroom has shown that children with ADHD attend to distractions on 25% of the trials that they miss, as compared to only 1% for the control group (Parsons et al., 2007). Adams et al. (2009) found that the virtual classroom classified ADHD more accurately than traditional CPTs (87.5% vs. 68.8%). Additionally, children rated the virtual classroom as more enjoyable than a typical CPT (Pollak et al., 2010). Thus, the virtual classroom has the potential to measure the ability of cognitive training to improve attention by approximating a scenario in which attention, working memory, and executive abilities can be measured in an environment comparable to what is actually experienced by the subject in the real world.

### Working Memory and ADHD

Working memory, a target of rehabilitation in various commercial cognitive training programs, has been identified as a core deficit of ADHD (Engelhardt et al., 2008; Rapport et al., 2008). As such, working memory may play a vital role in maintaining focused behavior in practical situations, making working memory training salient to efforts to improve classroom performance. For instance, Kane et al. (2007) found that individuals with lower working memory capacity endorsed significantly more mind wandering and off-task thoughts during cognitively demanding activities in everyday life than peers with stronger working memory capacity. Ramos et al. (2019) used performance on the digit span backward subtest to highlight verbal working memory as a key cognitive impairment in children with ADHD and revealed that deficits are more pronounced in younger children. Though children may be more functionally affected by working memory dysfunction, these deficits persist into adulthood (Alderson et al., 2013). Neurologically, the shared neural systems of working memory and attention are activated in the prefrontal cortex when information is maintained in one’s mind for a spatial attention task (Ikkai and Curtis, 2011). The capacity for working memory has been linked to reading (Dahlin, 2011) and math achievement (Geary et al., 2004), as well as later academic attainment in general (Gathercole et al., 2003). Moreover, the presence of working memory deficits in children with ADHD lead to worse functional outcomes and increase the risk for academic problems in comparison to children with ADHD without a working memory deficit (Fried et al., 2016). Thus, working memory as a cognitive skill may be a target in the broader treatment of ADHD.

### Computerized Working Memory Training

Evaluation of computerized interventions aimed at strengthening working memory has yielded mixed findings. For example, Beck et al. (2010) found computerized working memory training improved scores of attention and executive functioning, as well as parent ratings of impulsivity and hyperactivity. McNab et al. (2009) found that after working memory training, neural systems that are believed to underlie working memory exhibit increased activation and altered dopamine receptor binding. However, meta-analyses have tempered these initial findings with a more skeptical interpretation, citing the lack of consistent evidence for functional improvements after training (Sonuga-Barke et al., 2014; Cortese et al., 2015). Although research has consistently demonstrated training-related improvement on neuropsychological measures of working memory, Melby-Lervåg and Hulme’s (2012) and Shipstead et al.’s (2012) meta-analyses did not find generalizability of working memory training on far-transfer tasks.

Methodological problems, lack of valid real world assessment instruments, and the intervention’s uncertain mechanism of action are all challenges to outcome research. For instance, Chacko et al. (2013a, b) convincingly demonstrated the lack of posttraining improvement on tasks that do not significantly overlap with training tasks. In a compelling response to this study, Gathercole (2014) proposed new approaches to assess transfer effects and called for innovative methods to detect training-related behavior changes that are often difficult to evaluate.

### Improving Ecological Relevance

Widespread evidence of generalizable effects from cognitive training resulting in improvements in everyday life remains a focus of debate, though early findings demonstrated improvements in fluid intelligence (e.g., Jaeggi et al., 2008) and there is some evidence for the transfer of learning to reading comprehension, math performance, and attentional control (e.g., Holmes et al., 2009; Beck et al., 2010). Further, working memory training, when administered by teachers in a school setting, has been shown to improve general academic progress (Holmes and Gathercole, 2013).

With respect to ADHD, however, the effect on behavioral correlates in the classroom has been difficult to measure. The reliance on parent and teacher ratings as the primary tool to establish evidence for transfer effects (see Bigora et al., 2016) is problematic since they have been shown to be discrepant and of questionable validity (e.g., Cho et al., 2011). If working memory training is to be legitimized as an effective treatment, the use of ecologically valid outcome measurement to document efficacy is paramount; hence, the need to develop methods of measuring real-world working memory, such as how well one can carry out a complex list of instructions or sustain on-task behaviors in the classroom (Gathercole et al., 2008; Green et al., 2012). Whereas the development of novel working memory measurements to improve ecological relevance has been an objective of such research (Gathercole et al., 2008; Holmes et al., 2009), assessing cognitive functioning in a VR setting presents an opportunity for improved experimental control and may better capture training effects related to behavior in the classroom environment. VR assessment eliminates bias inherent in questionnaire assessment and potentially offers a psychometrically sound estimate of a child’s behavior during classroom tasks that demand sustained attention (Rizzo and Koenig, 2017).

### Hypothesis and Aims of the Study

The current study sought to introduce a novel, ecologically relevant attention task to capture classroom-related improvements in sustained attention and behavioral control after working memory training. Although evidence regarding the effectiveness of computerized working memory training can be gleaned from this study, the overarching aim was to demonstrate that changes in performance on real-world tasks after training can provide incrementally helpful and generalizable information about post-training functional improvements. We hypothesized that performance on the virtual classroom CPT would improve after 5 weeks of computerized working memory training.

## Materials and Methods

### Participants

The current study included 15 participants (12 boys and 3 girls) between the ages of 6 and 13 (*M* = 10.5 years; *SD* = 2.25). All were right handed. The average WISC-IV Full Scale IQ was 108.3 (*SD* = 15.7). The participants varied in ethnicity, with 53% endorsing Caucasian, 20% African American, and 27% Asian or Pacific Islander. Participants were initially recruited from the Emerging Needs program in a private elementary school, designed to identify and support the unique learning and attention needs of students. Recruitment was coordinated with the director of the program who provided information about the study to parents. Though a larger pool of participants were initially recruited, participants included in this study were screened for attention problems and potential diagnosis of ADHD via an attention questionnaire completed by both a parent and the child’s teacher. Despite the heterogeneous nature of this sample, which included a range of severity with respect to attention problems, all participants were receiving special accommodations through the emerging needs program. After exhausting this pool, a second phase of recruitment drew participants with similar attention problems from clinical networks in the community. Participants were not included in the study if they had been diagnosed with a previous or existing neurological or psychiatric disorder. Parental written informed consent and assent of each participant was obtained prior to enrollment in the study. Each family was provided personal feedback about their child via an abbreviated research report of findings based on neuropsychological testing performance. The current research was completed with approval from Fuller Graduate School of Psychology’s Institutional Review Board.

### Measures

#### Virtual Reality Classroom Experimental Task

**FIGURE 1:**
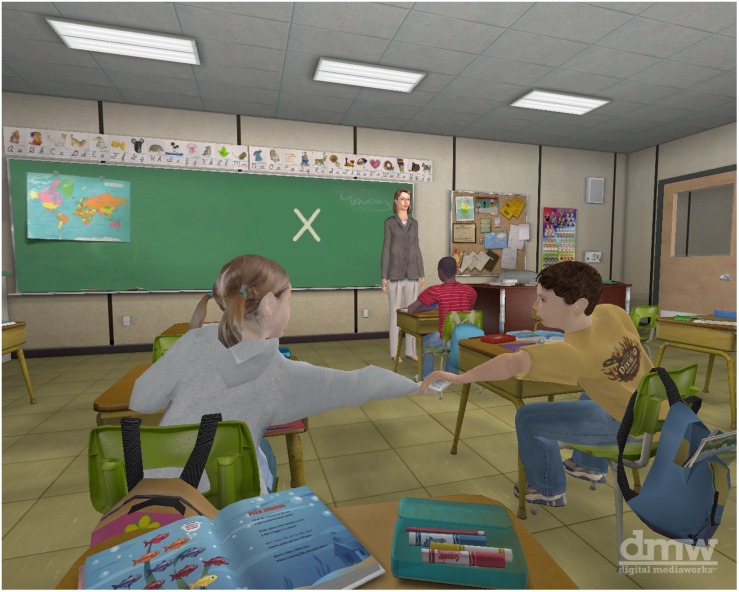
A screenshot from the virtual classroom CPT.

The virtual classroom was administered to all participants. The virtual classroom uses a virtual reality head mounted display (HMD) system for the assessment of attention processes, and is specifically designed to measure sustained attention, impulsivity, and distractibility (Nolin et al., 2016). The virtual classroom was used on a Pentium 4 level laptop computer with 1 GB RAM and a 128 MB DirectX 9-compatible NVIDIA 3D graphics card. The eMagin z800, with displays capable of 800 × 600 resolution within a 40-degree diagonal field of view, was the HMD used. Within the virtual classroom, participants find themselves sitting at a square desk in a traditional classroom containing adjacent rows of desks occupied by other students. There is a female teacher at the front of the classroom, a blackboard, and a large window to the left of the participant that looks out into a busy street (see Figure 1). Within the virtual environment, participants experience common classroom distractions that can be controlled and manipulated to approximate a life-like classroom setting.

Respondents are instructed to view a series of letters presented on the blackboard and to respond by hitting the spacebar on the keyboard only after observing an “X” preceded by an “A” and to withhold responding in any other condition. The letters were presented at a rate of one every 1350 ms and remained on the screen for 150 ms, with trials lasting 10 min, comprised of 400 stimuli. There were pure auditory distracters (classroom noises), pure visual distracters (paper airplane flying across the visual field), and mixed auditory and visual distracters (a car rumbling by the window and a person walking into the classroom with hall sounds occurring when the door to the room was opened). Each distracter was displayed for 5 s and presented in randomly assigned intervals of 10, 15, or 25 s. A total of 30 distracters (10 different distracters, three of each) were included in the 10-min scenario. Variables of interest included traditional CPT measurements such as omission errors, commission errors, reaction time, and reaction time variability. Head movement variables were represented by the number of degrees moved across each of three axes. Larger numbers indicate more head movement.

#### Traditional Working Memory Subtests

The Wechsler Intelligence Scale for Children, Fourth Edition (WISC-IV; Wechsler, 2003) core Working Memory subtests were used to assess working memory in a traditional way. Test-retest reliability coefficients for letter-number sequencing and Digit Span have been found to be 0.90 and 0.87, respectively. The total Working Memory Index, a composite, age-corrected score, has been found to have a reliability coefficient of 0.92 and Cronbach’s alpha of 0.71 (Wechsler, 2003).

#### Attention Questionnaire

Parent and teacher versions of the Conners Rating Scales, Third Edition (Conners 3; Conners, 2008) were used to quantify symptoms of ADHD, and common co-morbid problems. Items corresponding to the nine DSM-5 criteria for the inattentive and hyperactive presentations of ADHD were tallied, as were symptoms of conduct disorder, oppositional defiant disorder, anxiety, and depression. The Conners 3 ADHD Index provides a probability of an ADHD diagnosis.

#### Cogmed Index Improvement

The index improvement is an aggregate score related to progress on Cogmed exercises and is calculated by averaging performance on an individual’s three best training exercises over the 25-day training period. A difference score is calculated between the trainee’s highest and lowest daily score and represents the progress of the participant on trained working memory measures.

### Procedures

In Phase 1, participants were administered a pre-intervention neuropsychological evaluation consisting of a standardized traditional battery of instruments and the virtual classroom task in two sessions, for a total of up to 6 h of testing.

Directly following the initial assessment, participants and their parents met with a qualified Cogmed coach on the research staff to begin the Cogmed intervention. Cogmed employs a highly supportive training structure which includes feedback from a one-on-one coach and training aid to ensure compliance and motivation throughout training. Specifically, the Cogmed training method consists of 25 computerized training sessions, each 30–45 min long. Each session consists of a selection of various tasks that target the different aspects of working memory. The training program, completed via home computer, is 5 weeks long with five sessions every week. Weekly, the coach called the trainee and family to discuss progress and troubleshoot any problems. After completion of the training protocol, the coach conducted a final wrap-up session to summarize the training and provide feedback about progress. Several months later, participants were administered an assessment battery identical to that which was administered in Phase 1. There was an average of 9.2 months between testing in the two phases (*SD* = 5.2).

## Results

Although 15 participants completed the working memory subtests of the WISC-IV and pre- and post-assessments, 2 participants were not included in the virtual classroom data analysis due to non-compliance with task directions on the virtual classroom (they had extremely high numbers of commission errors, rendering the protocol invalid), and 2 participants were omitted from the working memory measure analysis due to missing data.

The 15 participants had an average of 6.4 (*SD* = 2.5) of the nine DSM-5 criteria for the inattentive presentation of ADHD, and an average of 4.3 (*SD* = 3.2) of the nine DSM-5 criteria for the hyperactive-impulsive presentation of ADHD. According to the Conners 3 ADHD Index, the probability of an ADHD diagnosis ranged from 51 to 99%, with an average of 79.9% 4 (*SD* = 18.4). Most (all but 4) had zero symptoms of a conduct disorder, whereas 10 had at least one symptom of an oppositional defiant disorder, with an average of 3.0 symptoms (*SD* = 2.3) for those who had symptoms. Symptoms of anxiety or depression affected 12 of the 15 participants to some extent. For all but two of the participants, problems were rated as often or very often seriously affecting schoolwork or home life or friendships and relationships.

### Virtual Reality Attention Measures

**FIGURE 2:**
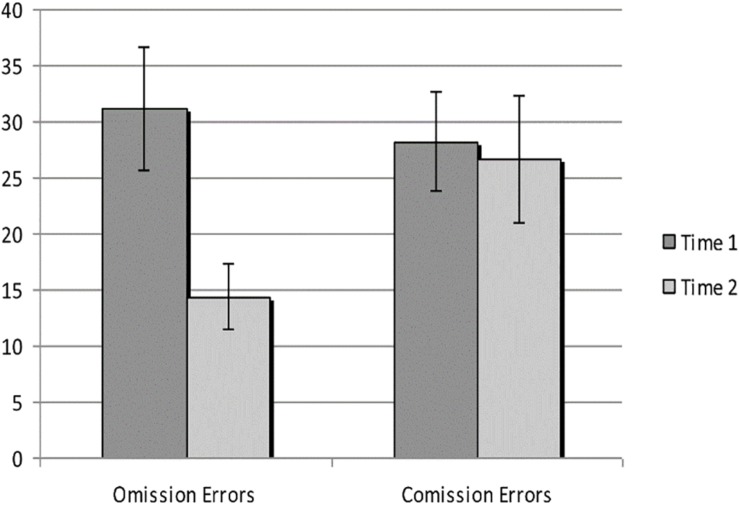
Omission errors and commission errors on virtual classroom CPT at Time 1 and Time 2.

To address the hypothesis, paired-samples *t* tests were conducted to examine differences in various aspects of performance in the virtual classroom between pre- and post-assessments. Figure 2 illustrates the differences in omission and commission errors before and after the intervention.

Results of paired-samples *t* tests showed significant improvement in the number of omission errors, hit variability, reaction time, and reaction time variability, as can be seen in Table 1. There was a trend toward improvement in sensitivity, *t*(12) = 2.03, *p* = 0.06. Differences in head movements were not found to be significant.

**TABLE 1:** Mean differences on virtual classroom CPT, Time 1, and Time 2.

**Variable**	**Time 1 *(n* = 13)**		**Time 2 *(n* = 13)**		***t(12)***	***d***
	***M***	***SD***	***M***	***SD***		
Omission errors	31.15	19.84	14.38	10.70	3.49^∗∗^	1.05
Hit variability	3.09	1.92	1.57	0.95	3.77^∗∗^	1.00
Reaction time (in seconds)	0.49	0.07	0.44	0.06	3.62^∗∗^	0.58
Reaction time variability	0.18	0.06	0.15	0.05	3.14^∗∗^	0.54
Commission errors	28.23	15.90	26.69	20.76	0.25	ns
RT to commissions	0.51	0.08	0.50	0.10	0.20	ns
Commission variability	0.24	0.07	0.24	0.13	0.04	ns
A’	0.79	0.13	0.86	0.11	2.02	ns
H’	–0.46	1.26	–2.06	4.97	1.13	ns
HM yaw range	5.04	3.47	3.17	3.49	1.35	ns
HM pitch range	7.56	5.13	8.01	7.03	0.18	ns
HM tilt range	3.94	3.10	4.55	4.84	0.35	ns

*RT, reaction time; A’, measure of sensitivity; H’, measure of response style; HM, head movements recorded by head-mounted display; ns, not significant. ^∗∗^*p* < 0.01.*

### Working Memory Measures

We averaged the index improvement recorded for each participant which at 25.4 units (*SD* = 9.11) was similar to that of the standardized sample (27 units). The sample’s index improvement was also negatively correlated with age, *r*(12) = −0.63, *p* < 0.05.

**FIGURE 3:**
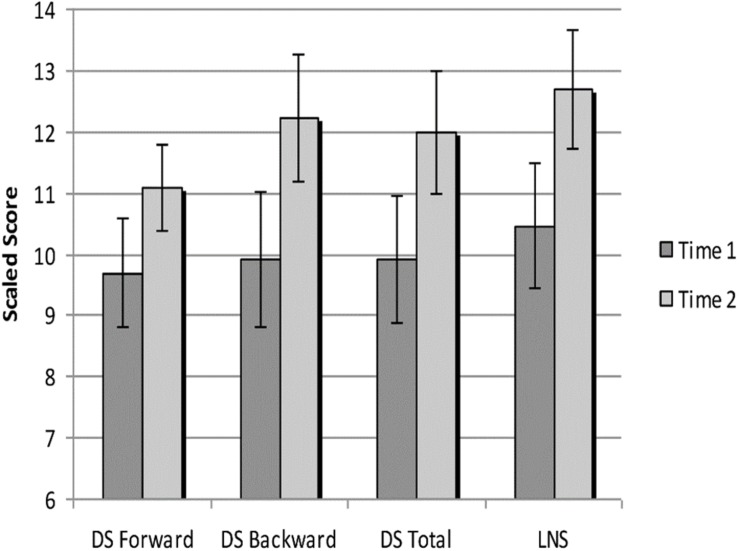
Performance on WISC-IV working memory index subtests at Time 1 and Time 2. Scores are represented as age-corrected scaled scores. DS, digit span; LN, letter number sequencing.

To confirm expected near-transfer effects, performance on WISC-IV working memory subtests were analyzed using paired-samples *t* tests, as can be seen in Table 2. Scaled scores for Digit Span Backward increased from 9.9 (*SD* = 4.0) to 12.2 (*SD* = 3.7), *t*(12) = 2.38, *p* < 0.05, and for letter-number sequencing from 10.5 (*SD* = 3.7) to 12.7 (*SD* = 3.5), *t*(12) = 2.67, *p* < 0.05. Scaled scores for WISC-IV Digit Span Forward trended toward improvement, from 9.7 (*SD* = 3.2) to 11.1 (*SD* = 2.6), *t*(12) = 1.6, *p* = 0.13. Moderate effect sizes were observed ranging from Cohen’s *d* = 0.56 to *d* = 0.72. See Figure 3 for a graph of these improvements.

**TABLE 2:** Mean differences on WISC-IV working memory subtests between Time 1 and Time 2.

**Variable**	**Time 1 *(n* = 13)**		**Time 2 *(n* = 13)**		***t(12)***	***d***
	***M***	***SD***	***M***	***SD***		
DS forward scaled score	9.69	3.20	11.08	2.56	1.64	*ns*
DS backward scaled score	9.92	4.01	12.23	3.74	2.38^∗^	0.60
DS total raw score	14.62	4.27	17.69	4.31	3.33^∗∗^	0.72
DS total scaled score	9.92	3.77	12.00	3.62	2.44^∗^	0.56
LN raw score	15.92	4.87	18.62	3.25	3.09^∗∗^	0.65
LN scaled score	10.46	3.69	12.69	3.54	2.67^∗^	0.62
WMI composite score	100.54	19.42	111.23	16.49	3.04^∗∗^	0.59

*DS, digit span; LN, letter-number sequencing; WMI, working memory index. ^∗^*p* < 0.05; ^∗∗^*p* < 0.01.*

Tables 3, 4 provide correlations between demographic variables and WISC-IV working memory measures. In general, variables such as age, time elapsed between testing, IQ, and progress on the working memory training intervention were not correlated with WISC-IV working memory changes, with the exceptions of IQ and the time elapsed between pre- and post-intervention testing sessions, which were associated with the improvement in letter-number sequencing scores as can be seen in Table 3. Children who scored higher on WISC-IV full-scale IQ displayed less the improvement in letter-number sequencing, and the longer the time between pre- and post-intervention testing, the stronger the improvement in letter-number sequencing.

**TABLE 3:** Correlations of demographic variables and working memory subtest difference scores.

**Variable**	**Gender**	**FSIQ**	**Time**	**Age 1**	**Age 2**	**II**	**DSB**	**DST**	**LN**	**WMI**
**Gender**										
FSIQ	–0.23									
Time between testing	0.09	–0.52								
Age (Time 1)	0.22	–0.02	–0.45							
Age (Time 2)	0.26	–0.15	–0.25	0.98^∗∗^						
Index improvement	−0.65^∗^	0.13	0.36	−0.58^∗^	–0.55					
DS backward raw score	0.08	0.08	0.15	–0.31	–0.30	0.35				
DS total raw score	–0.13	–0.18	0.05	–0.22	–0.23	0.45	79^∗∗^			
LN raw score	–0.19	−0.59^∗^	0.60^∗^	–0.19	–0.06	0.40	0.07	0.14		
Working memory index	–0.22	–0.45	0.15	0.01	0.05	0.46	0.50	77^∗∗^	0.61^∗^	

*Time, time between testing; II, index improvement; DSB, digit span backward; DST, digit span total; LN, letter-number sequencing; WMI, working memory index. Male participants achieved significantly great index improvement in comparison to females (28.2 vs. 15 units); a statistically significant difference, *t*(15) = 3.3, *p* < 0.1. ^∗^*p* < 0.05; ^∗∗^*p* < 0.01.*

**TABLE 4:** Correlations between demographic variables and virtual classroom difference scores.

**Variable**	**Gender**	**FSIQ**	**Time**	**Age 1**	**Age 2**	**II**	**Om**	**RT**	**HV**	**RTV**
**Gender**										
FSIQ	–0.28									
Time between testing	0.30	–0.23								
Age (Time 1)	0.04	–0.22	−0.57^∗^							
Age (Time 2)	0.09	–0.27	–0.46	0.99^∗∗^						
Index improvement	−0.60^∗^	0.50	0.07	−0.59^∗^	−0.63^∗^					
Omission errors	–0.29	−0.61^∗^	0.19	–0.07	–0.05	0.28				
Reaction time	0.11	–0.05	0.48	–0.03	0.04	0.28	0.31			
Hit variability	–0.43	–0.56	–0.24	−0.55^∗^	−0.56^∗^	–0.17	−0.58^∗^	0.05		
Reaction time variability	–0.27	–0.10	0.32	–0.20	–0.16	0.47	0.42	0.57^∗^	0.21	

*Time, time between testing; II, index improvement; Om, omission errors; RT, reaction time; HV, hit variability; RTV, reaction time variability. ^∗^*p* < 0.05. ^∗∗^*p* < 0.01.*

## Discussion

To our knowledge, no other researchers have attempted to measure working memory training effects by approximating real world functioning in a controlled virtual environment. Results confirmed our hypothesis that capturing training effects by means of VR assessment of classroom-related attention can imply far-transfer effects and offer incremental validity in the evaluation of training efficacy. Improvement observed on post-training traditional working memory measures was expected and consistent with the literature. A main implication of these findings is that psychometrically sound measurement tools are available and essential in determining functional outcomes of cognitive training and research should no longer solely rely on estimates or samples of behavior on traditional paper and pencil tests. The virtual classroom CPT integratively meets two needs: (a) to have empirical support for training effects via standardized, psychometrically valid outcome measurement, and (b) to demonstrate that training effects are evident outside a laboratory research setting.

Significant mean improvements were observed on both virtual classroom measures of sustained attention, as well as traditional working memory and attention measures from pre- to post-training, suggesting that working memory training not only improved working memory capacity but also generalized to sustained attention. Thus, it can be inferred that children were better able to resist distractions and maintain focus on the target stimuli as a result of the training, consistent with research that shows working memory capacity to be linked to the ability to resist distraction from irrelevant stimuli (de Fockert et al., 2001). Because the virtual classroom offers embedded distractions that closely resemble those in real life, finding post-training improvement in this domain should be intriguing to educators and interventionists. Reaction time also improved, suggesting an improvement in processing speed. An effect of general video game-style cognitive training on processing speed has been demonstrated and is expected considering the design of the training intervention that rewards quickly responding to stimuli (see Nouchi et al., 2013). Additionally, children improved consistency in both accuracy and speed of responding as measured by hit variability and reaction time variability. These findings are important due to the known characteristics of ADHD performance on CPT tasks: Typical response patterns show a decline in the percentage of correct responses and reaction time as a function of time from start to finish (Epstein et al., 2003). As this performance pattern is a distinct feature of ADHD, improvement on this task in an ecologically relevant environment is promising. Interestingly, the present results did not yield significant differences in head movements during the sustained attention task, contrary to Klingberg et al. (2002)’s finding of a reduction in head movements during the assessment of children with ADHD after working memory training.

Considering the mechanisms for transfer, the shared neural systems between working memory and attention may explain the observed near-transfer effect to attention (Oleson et al., 2004; Ikkai and Curtis, 2011). It is conceivable that training-induced plasticity in working memory areas of the brain also yields plasticity-related improvements in attention performance. Alternatively, training may improve cognitive processes that support attention functioning. As noted by Holmes et al. (2010), the intense and prolonged nature of the intervention may encourage the development of working memory strategies that compensate for weaknesses in basic processes. Anecdotally, participants in our study tended to report using such acquired strategies in everyday life. Though the development of task-specific strategies, theoretically, should be applied only to tasks similar to training, the virtual classroom may provide a unique opportunity to employ these acquired functional skills.

Finally, the cognitive abilities employed in the virtual classroom represent a much broader transfer of learning than traditional neuropsychological measures, and thus improve generalizability. In this way, our findings build on the meta-analysis by Spencer-Smith and Klingberg (2015), who generally defined “inattention in daily life” by parent or teacher ratings in their assertion that working memory training does, indeed, lead to functional improvements in daily life.

### Limitations

In addressing limitations, it must be emphasized that the current research does not seek to establish the efficacy of specific cognitive training programs but, rather, to further the conversation about ecologically relevant outcome measurement. As others have called for higher standards of outcome measurement and espoused significant skepticism about claims of far transfer effects, this research aims to orient the field toward the viability of VR assessment.

A major limitation of the current study is the lack of comparison group with which to compare post-training outcomes on the virtual classroom and beyond. Without this baseline control, it is difficult to evaluate whether improvements on post-testing were genuinely related to training effects or, rather, other developmental, environmental, or pathognomonic factors. A practice effect across testing time points was not believed to contribute to improvement on non-trained measures of attention given that CPT tasks are generally considered to have strong test-retest reliability and to be relatively unaffected by practice effects (see Conners et al., 1998). It will be important to substantiate these findings with an age-corrected control group or a normative sample once standardized norms are available for the virtual classroom in new, technologically advanced iterations.

Another major limitation of the current study is the small size and heterogeneous nature of the sample which poses challenges to interpretation of the data. Clinical data on the participants suggested some level of co-occurring disorders, specifically depression and anxiety. With the high rate of comorbidity between ADHD and depression and anxiety, some level of co-occurrence should be expected (Schatz and Rostain, 2006).

### Conclusion and Future Directions

The present study showed assessment within a virtual environment can provide incremental validity for the effectiveness of the intensive and adaptive training of working memory, and how such environments may give unique opportunity to measure transfer effects to associated cognitive domains including attention. The primary implication of this main finding is the usefulness of a unique and ecologically relevant measurement tool to aid in the evaluation of new treatments for ADHD and learning disabilities. Most salient is the need for future research to analyze authentic training-related improvements using a randomized, placebo-controlled research design. An increased sample size of participants, smaller age range, and less variability in terms of psychiatric symptoms would also allow for conclusions about training efficacy. The growing field of computerized cognitive intervention is looking to novel methods of studying important developmental, cognitive, and learning constructs that closely resemble behavior in the real world. The virtual classroom offers one such novel measure. With rapid advances in the affordability, portability, and quality of VR experiences, the technology is ready to be meaningfully incorporated into clinical and educational settings and can meet a critical need to scrupulously appraise the value of cognitive training. Without the generalizability limitations of traditional paper and pencil assessment, ecologically relevant assessments such as the virtual classroom may help answer a crucial question; does working memory training truly improve a child’s ability to stay on task in the classroom?

## Data Availability

The datasets generated for this study are available on request to the corresponding author.

## Ethics Statement

This study was carried out in accordance with the recommendations of the Institutional Review Board at Fuller Graduate School of Psychology with written informed consent from all subjects. All subjects gave written informed consent in accordance with the Declaration of Helsinki. The protocol was approved by the Travis Research Institute.

## Author Contributions

BC was the main author of the manuscript, and the original work has been taken from the doctoral dissertation. SM was the principal investigator on the study and dissertation chair, and co-writer of the manuscript. AR was the project partner, supported on all the VR aspect, interpreted the VR data, and revised and edited the manuscript. JT was co-principal investigator on the study, designed the study, and drafted the original work as dissertation committee. AN provided the major contributions to the study design, project management, interpretation of data, and co-writing of the manuscript.

## Conflict of Interest Statement

The authors declare that the research was conducted in the absence of any commercial or financial relationships that could be construed as a potential conflict of interest.
